# Nanopipette exploring nanoworld

**DOI:** 10.1186/s40580-014-0017-3

**Published:** 2014-04-25

**Authors:** Tomohide Takami, Bae Ho Park, Tomoji Kawai

**Affiliations:** Division of Quantum Phases and Devices, Department of Physics, Konkuk University, Seoul, 143-701 Korea

**Keywords:** Nanopipette, Ion-selective electrode, Scanning ion conductance microscopy, Sodium, Potassium

## Abstract

Nanopipettes, with tip orifices on the order of tens to hundreds of nanometers, have been utilized in the fields of analytical chemistry and nanophysiology. Nanopipettes make nanofabrication possible at liquid/solid interfaces. Moreover, they are utilized in time-resolved measurements and for imaging biological materials, *e.g.*, living cells, by using techniques such as scanning ion-conductance microscopy and scanning electrochemical microscopy. We have successfully fabricated ion-selective nanopipettes that can be used to identify targeted ions such as sodium and potassium in- and outside of living cells. In this review, we discuss the extent of utilization of nanopipettes in investigating the nanoworld. In addition, we discuss the potential applications of future nanopipettes.

## Introduction

Barber used a glass micropipette as an intracellular microelectrode in 1902 [1]. Since then, micropipettes have been a useful tool in biology, especially for cell physiology. They have been used for the delivery of genes and sperms to target cells including ova [2–5]. A more famous application of micropipettes is their use in the patch-clamp method by Neher and Sakmann in 1976, detecting voltages and currents from ion-channels [6]. Micropipettes have been used mainly in the field of cell biology for delivering materials or detecting physiological signals.

**Figure 1:**
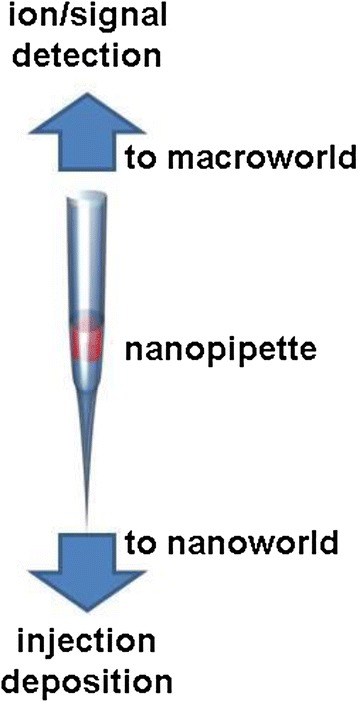
**Concept of a nanopipette: a bridge between macro- and nano-world.**

Recently, the demand for sensitive analytical and diagnostic tools to identify biomolecules has increased in the field of biosensing and biomedicine [7]. Nanopipettes can play the role of a bridge connecting the macro- and nano-world, as shown in Figure 1. They can transfer a liquid in volumes less than a zeptoliter [8], as well as can transfer small amounts of materials such as nanoparticles, chemical compounds, and proteins. In addition, they can receive signals of voltage, current, and ions. Thus, nanopipettes are useful tools for cutting-edge nanotechnology, especially in the field of bionanotechnology [9].

Nanopipettes are also used for imaging materials, especially soft materials, in liquids. Bard et al. invented scanning electrochemical microscopy (SECM) [10], in which faradaic current between an electrode placed in a nanopipette and a sample due to an electrochemical reaction is probed and the feedback is applied in the same way as in electrochemical scanning tunneling microscopy [11]. Hansma et al. invented scanning ion conductance microscopy (SICM) [12], in which ionic current between an electrode placed in a nanopipette and a sample is probed but the opposite feedback is applied since the ionic current decreases with a decrease in the distance between the nanopipette top and sample. The imagings with SECM and SICM involving the use of a nanopipette have been reviewed in several papers [13–17]. Glass nanopipettes with an outer diameter of less than 1 μm can be fabricated using a puller --same apparatus used for fabricating micropipettes but with different fabrication parameters. Boron silicate nanopipettes are commonly fabricated for many purposes. Though the fabrication of quartz nanopipettes is more preferable than that of boron silicate nanopipettes owing to the reproducibility of fabricating nanopipettes with various shapes and diameters less than 100 nm and the significant advantage of lower leakage currents [18], quartz nanopipettes with diameters less than 50 nm are easily chipped when a transverse shear force is applied at their top. Glass nanopipettes are used mainly in the field of analytical chemistry and several review papers are available on this topic [18–21].

In this short review, we briefly discuss the applications of nanopipettes—ranging from the transfer of materials to detection of ions and voltage/current signals. In the last section, we present our perspective on the future applications of nanopipettes, based on recent studies.

## Review

### Nanofluidics in nanopipettes

Nanofluidics is a hot research topic these days and is related to applications such as single molecule deoxyribonucleic acid (DNA) sequencing using nanopores [22]. Several good review papers [23–26] as well as a theory paper [27] are available on nanofluidics. In this chapter, we discuss the anomalous nanofluidic phenomena that occur in nanopipettes.

Glass nanopipettes respond to a symmetric voltage input by exhibiting asymmetric output current, an effect termed ion current rectification (ICR) [28]; this behavior of glass nanopipettes is significantly different from that of conventional micropipettes. Ion current rectification is attributed to the formation of a diffuse electrical double layer within the nanopipette tip orifice. When the double layer thickness is comparable to the nanopipette diameter, the electrostatic interaction between an ionic species and surface charges will affect ion transport properties [7]. The electrostatic interaction has been shown to be influenced by electrolyte concentration, pH, and applied voltage, and can be modulated by functional layers deposited or covalently attached to the nanopipette mouse. Poly-L-lysine, a polypeptide bearing positively charged amino groups, can be physisorbed on a negatively charged nanopipette surface [29], and the protonated amino groups invert current rectification. Similar results were observed in case of a nanopipette modified with cationic dendrimers [30] and in a protein-binding study on PEG-modified nanopipette-like structures [31]. Recently, we reported the dependence of ICR on the concentration gradient of KCl solutions in polyethyleneimine-modified glass nanopipettes [32]. The peak shape of the rectification factor was found to depend on the outer KCl solution concentration when the inner KCl solution concentration ranged from 1 mM to 500 mM. Such peak shape dependence was also observed when the concentrations of the inner and outer KCl solutions were controlled. The peak shape of the ICR curve could be attributed to ion conductance changes through the conical nanopipette, which result from modulation of ion concentration.

**Figure 2:**
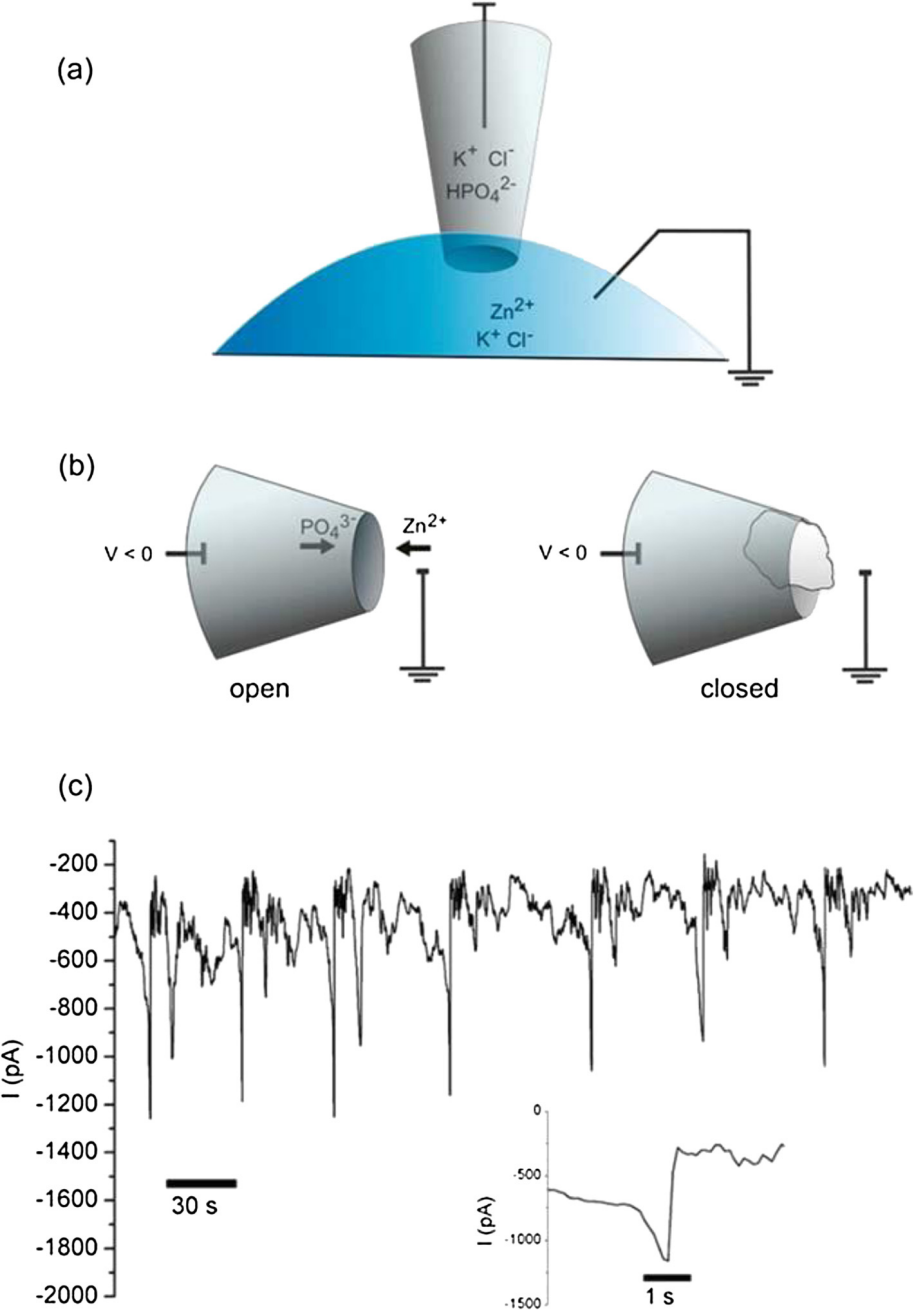
**Ion current oscillations in a nanopipette induced by nano-precipitation. (a)** Electrochemical setup to measure ion current through a quartz nanopipette. All solutions contain 0.1 M KCl and are buffered at pH 7, with 10 mM potassium phosphate in the barrel and 10 mM Tris–HCl in the bath. Zinc chloride is included in the bath at concentrations of 2--20 μM. **(b)** Configuration causing ion current oscillations. A negative potential in the nanopipette barrel draws zinc cations from the bath into the pore, while phosphate ions are pushed out into the bath. When a precipitate of sufficient size is formed, the pore is blocked and ionic current decreases. **(c)** Current oscillations in a nanopipette setup with 2 μM zinc chloride in the bath and a potential of 350 mV. Inset: expanded view of one of the open states. From Ref. [37], copyright@2011 American Chemical Society.

The oscillation of ion current through a nanopipette is also an interesting phenomenon that might be similar to the non-linear behavior of signal transduction in the human brain [33]. Ion current oscillations were observed in rectifying conical nanopores in polyethylene terepthalate films, and were attributed to dynamic precipitation in the pore caused by voltage-induced concentration of weakly soluble salts [34,35]. Current oscillations in much larger pores of silicon nitride or borosilicate glass can be generated at the interface of two solvents by using organic molecules with differential solubility [36]. These phenomena offer a new way for electrically monitoring non-equilibrium events such as precipitation in real time and on the nanoscale. Vilozny et al. [37] reported on the phenomenon of voltage-dependent current oscillations arising from the precipitation of zinc phosphate, a salt with extremely low water solubility, at the pore of a quartz nanopipette (Figure 2). Further, we discovered a new phenomenon of ion current oscillations in a nanopipette with a different mechanism without precipitation: oscillations of the double layer thickness due to the adsorption and desorption of protons on the inner nanopipette surface resulted in nonlinear current oscillations at 2.7 mHz [38]. These nonlinear current oscillations without precipitation can be utilized for the fabrication of non-linear fluidic devices [39].

### Biosensors based on nanopipettes

Nanoscale electrochemical biosensors offer new scope and opportunities in analytical chemistry because the reduction in the size of electrochemical biosensors to nanoscale dimensions expands their analytical capability, allowing the exploration of nanoscopic domains, measurements of local concentration profiles, *in vivo* detection of various phenomena in microfluidic systems, *e.g.*, monitoring of neurochemical events via the detection of stimulated dopamine release [40]. Nanopipettes can be utilized for such various usages of biosensors. Indeed, nanopipettes have been used to obtain high-resolution topographic images of live cells under physiological conditions, and to create nanoscale features via controlled delivery of biomolecules [9].

Some of the studies on the biosensing applications of nanopipettes are summarized in a review paper by Actis et al. [7]. In another study, they showed that signal transduction by ion nano-gating sensors functionalized with aptamers allowed the quantitative detection of thrombin, and the binding of thrombin generated a signal directly correlated to its concentration in the bulk solution [41].

Sa et al. [42] examined dihydroimidazole-modified nanopipettes and the current--voltage response of these nanopipettes to pH changes and divalent metal ions. By examining the ICR response of nanopipettes, they observed a qualitative picture of the surface charge on the nanopipettes. They reported the observation of cobalt binding and regeneration of binding sites. In addition, they detected specific DNA sequences using ICR and dendrimer-modified nanopipettes [43]. They observed selective responses toward complementary and non-complementary sequences, as well as enhanced response relative to single-base mismatches toward perfectly complementary sequences.

Piper et al. [44] used a simply fabricated nanosensor to detect both ratiometric and intensity-based fluorescent changes for physiological levels of pH and sodium, respectively. Their method should be generally applicable to any fluorescence-based reporter dye and can thus be used for a wide range of analytes and concentrations. Further, their nanosensor opens up the possibility of being used for the nanoscale mapping of analytes over living cells.

Umehara et al. [45] demonstrated that electrostatic, biotin--streptavidin, and antibody--antigen interactions on the nanopipette tip surface affect ionic current flowing through a nanopipette pore. They also found that highly charged polymers interacting with the glass nanopipette surface modulated the rectification property of the nanopipette electrode. Affinity-based binding between probes tethered to the surface and target proteins caused a change in the ionic current owing to a partial blockade or an altered surface charge. Thus, their findings show that nanopipettes functionalized with appropriate molecular recognition elements can be used as nanosensors in biomedical and biological research.

Vitol et al. [46] reported an analytical approach to intracellular chemical sensing that utilizes a surface enhanced Raman spectroscopy (SERS)-enabled nanopipette. Their probe comprised of a glass capillary with a 100–500 nm tip coated with gold nanoparticles. The fixed geometry of these nanoparticles overcame the limitations of the traditional approach for intracellular SERS in which metal colloids were used. They demonstrated that SERS-enabled nanopipettes can be used for the *in situ* analysis of living cell functions in real time. In addition, the SERS functionality of the probes allowed the tracking of their localization in a cell. However, decorated structures outside the nanopipettes easily collapsed during the insertion and extraction processes.

**Figure 3:**
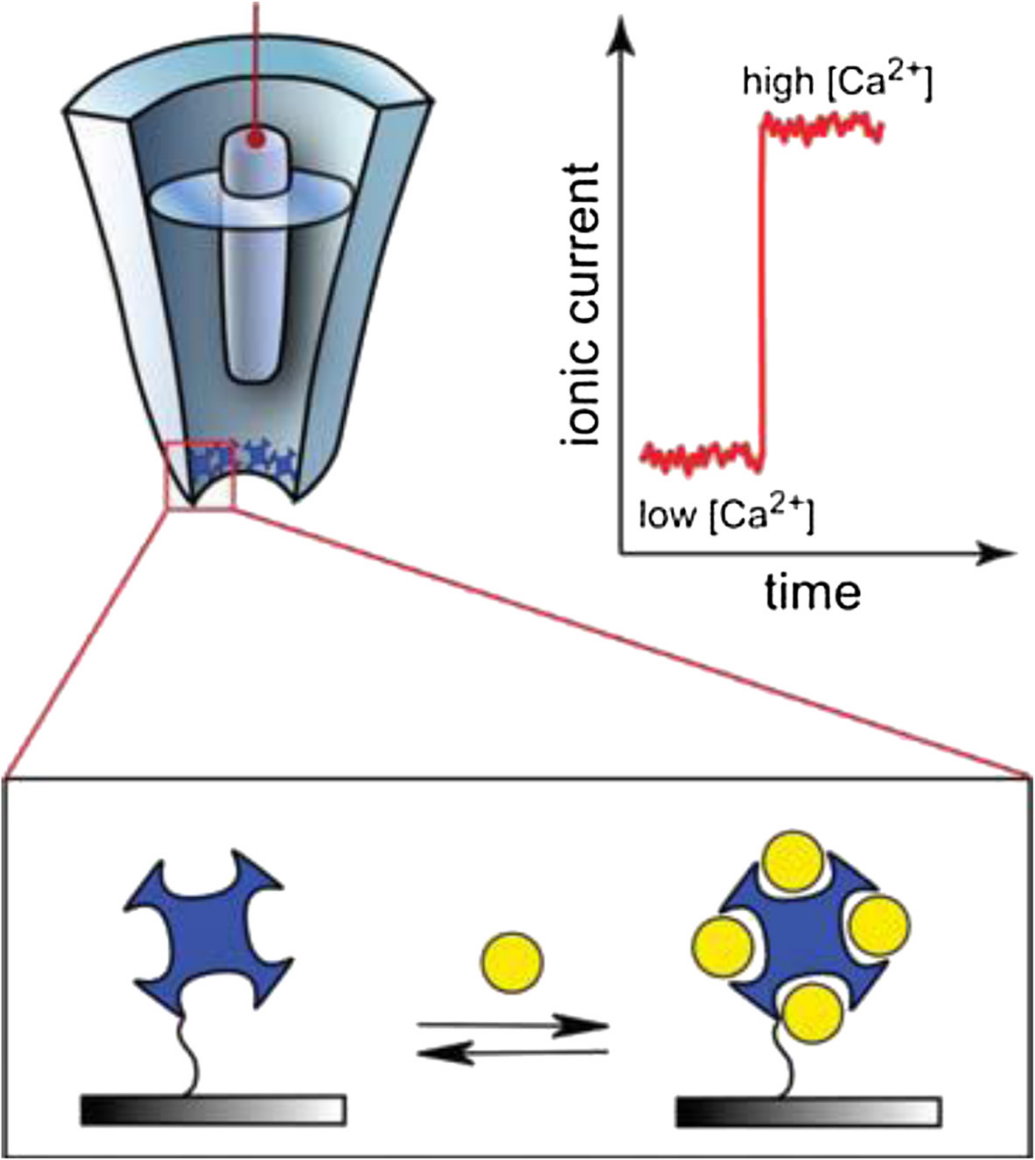
**Schematic of reversible calcium ion binding at the tip of a nanopipette.** As calcium ions (yellow spheres) are bound by calmodulin protein (blue), changes to the surface charge at the tip will affect the ion current. From Ref. [47], copyright@2011 American Chemical Society.

Vilozny et al. [47] fabricated a calmodulin-modified nanopipette that demonstrated an enhanced response to calcium ions, the binding of which was rapidly reversible (Figure 3). They obtained highly stable signals that enabled multiple measurements with a single nanopipette, thereby allowing the determination of binding constants. Improving the above-mentioned methods will enable the characterization of proteins confined in nanochannels, as well as the measurement of protein-protein interactions by using nanopore sensors. Reversible sensors based on nanopipettes may be used for the nanoscale spatial resolution of ion concentration and continuous intracellular measurements of specific analytes.

### Nanopipettes for materials transfer

In this chapter, the use of nanopipettes for transferring nanoscale materials is discussed. The most common mode of injection with a pipette is grouting with pressure. However, it is difficult to control a small amount of the injected material with a micropipette [48]. Moreover, it is impossible to transfer materials with a nanopipette by applying a high pressure. The required force to draw a solution inside a nanopipette can be obtained by applying a suitable voltage between an electrode placed in the nanopipette and another one placed in the outer solution, across the liquid-liquid interface. The solution can then be injected into the sample such as a biological cell [49].

**Figure 4:**
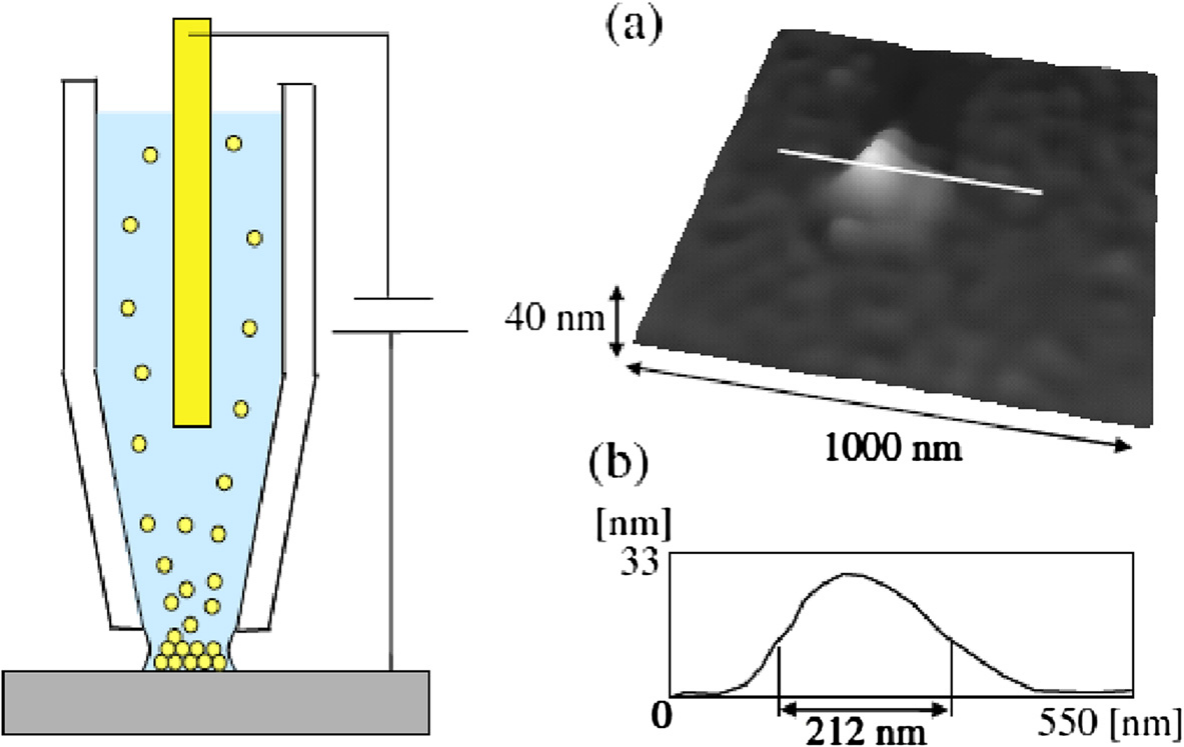
**Fabrication of a gold island consisting of nanoparticles by the electrophoretic deposition method with a nanopipette. (a)** Topographic image of the gold island deposited at a voltage of 30 V for 4 s. **(b)** Cross-sectional profile of the island structure shown in image **(a)**. From Ref. [50], copyright@2007 IOP Publishing.

Iwata et al. successfully made nano-dots using a nanopipette, as shown in Figure 4 [50]. A pipette-based electrochemical method allows deposition control via electrical delivery of materials in and out of the nanopipette. The advantages provided by this method include unique deposition modes realized using high electric fields and complicated pressure injection due to high resistance at the nanopipette tip. Further, they successfully fabricated a gold nanoparticle pattern by using a scanning nanopipette microscope equipped with a nanopipette probe filled with a colloidal solution, as shown in Figure 4. Suryavanshi and Yu developed the electrochemical fountain pen nanofabrication method and used it for local electrochemical deposition of high-quality and high-aspect-ratio freestanding copper [51] and platinum [52] nanowires. Further, Ito and Iwata [53] fabricated copper nanodots, and obtained a resolution almost equal to that reported by Suryavanshi and Yu [51].

Nano-fabrication using a nanopipette has been conducted by many researchers. Bruckbauer et al. demonstrated that it is possible to obtain a nanoarray with specific features having a diameter of 300 nm by the selective nanopipette delivery of a functional antibody [54]. This result is a significant improvement over the spot size achieved by direct nanopipette delivery onto a flat surface (800 nm) [55]. In addition, they developed a new nanopipette-based method for the controlled voltage-driven delivery of individual fluorescently labeled probe molecules to a plasma membrane to be used for single-molecule fluorescence tracking. Saslau et al. [16] reported in their review that the direct fabrication method of 2D and 3D polymer nanowires by using a nanopipette can also be utilized for nanoscale wire bonding.

A summary of noteworthy reports on the use of nanopipettes for deposition or patterning is presented in Table 1. Nanofabrication using nanopipettes can be employed not only for the deposition of metals but also for the injection of biomolecules such as DNA and proteins [56,57]. Recently An et al. combined the nanopipette injection system with tuning fork atomic force microscopy (AFM) to achieve a breakthrough in controlled nanomaterial delivery and selective deposition [58]. Funabashi et al. [59] demonstrated the feasibility of the femtoinjection of a decoy oligodeoxynucleotide into the nucleus of a single embryonic stem (ES) cell to suppress the activity of a transcription factor. Improvements in nanofabrication and nanoinjection methods using a nanopipette are being reported frequently.

**Table 1:** **Potential controlled dispensing with nanopipettes**

**Purpose/application**	**Deposited/delivered**	**Size or amount**	**Reference**
Metal growth	Au	212 nm width 30 nm height	[50]
	Pt	150 nm diameter	[51]
	Cu	200 nm diameter	[52–53]
	Ag	80 nm width line	[58]
Cellular injection	DNA/protein	< 100 fL	[49]
ES cell injection	Oligodeoxynucleotide	30 fL	[59]
Surface patterning	Antibody/antigen	300 nm	[54]
	DNA	830 nm	[56]
Pulsed delivery	DNA	150 molecules	[55]
Biochemical activation	Na^+^/OH^−^	1−30 μm	[57]

### Nanopipettes for imaging

The method of combining pipette probes with a microscope was developed by Bard for SECM [10] and Hansma for SICM [12]. Nanopipettes offer a novel approach for the study of membrane biology in which individual proteins can be imaged without the need for fluorescent labels [18].

In SECM, a nanoelectrode scans the region above a cell surface to measure faradaic currents associated with oxidation or reduction of electroactive compounds or freely diffusing ions in buffers [60]. As the radius of a nanoelectrode is thousand times smaller than that of a cell, the nanoelectrode easily penetrates a cell without any apparent damage to the membrane. This technique has been successfully exploited to measure transmembrane charge transport, to evaluate membrane potential and to probe subcellular redox properties at a spatial resolution of 200 nm.

Scanning ion conductance microscopy is a special type of scanning probe microscopy (SPM) that has been successfully adapted for imaging living cells at a nanoscale resolution [61]. A glass nanopipette is used to scan the sample surface and the nanopipette–sample distance is opposite-feedback controlled by the ion current flowing through the nanopipette without any physical contact. The resolution of SICM is determined by the inner radius of the nanopipette. The drawback of conventional raster-scanning SICM is that it is prone to probe sample collisions, and is limited to the imaging of relatively flat surfaces. Hopping mode SICM overcomes the constraint for noncontact nanoscale imaging, even on the most convoluted surface structures. In this mode, the nanopipette approaches a surface to measure heights only at selected imaging points where the current has a fixed value, around 1% of the reference current. The nanopipette is then rapidly withdrawn to a safe distance before moving on to the next imaging point. This hopping-mode technique can be used to visualize complex three-dimensional structures such as that of mechanosensitive stereocilia on auditory hair cells.

**Figure 5:**
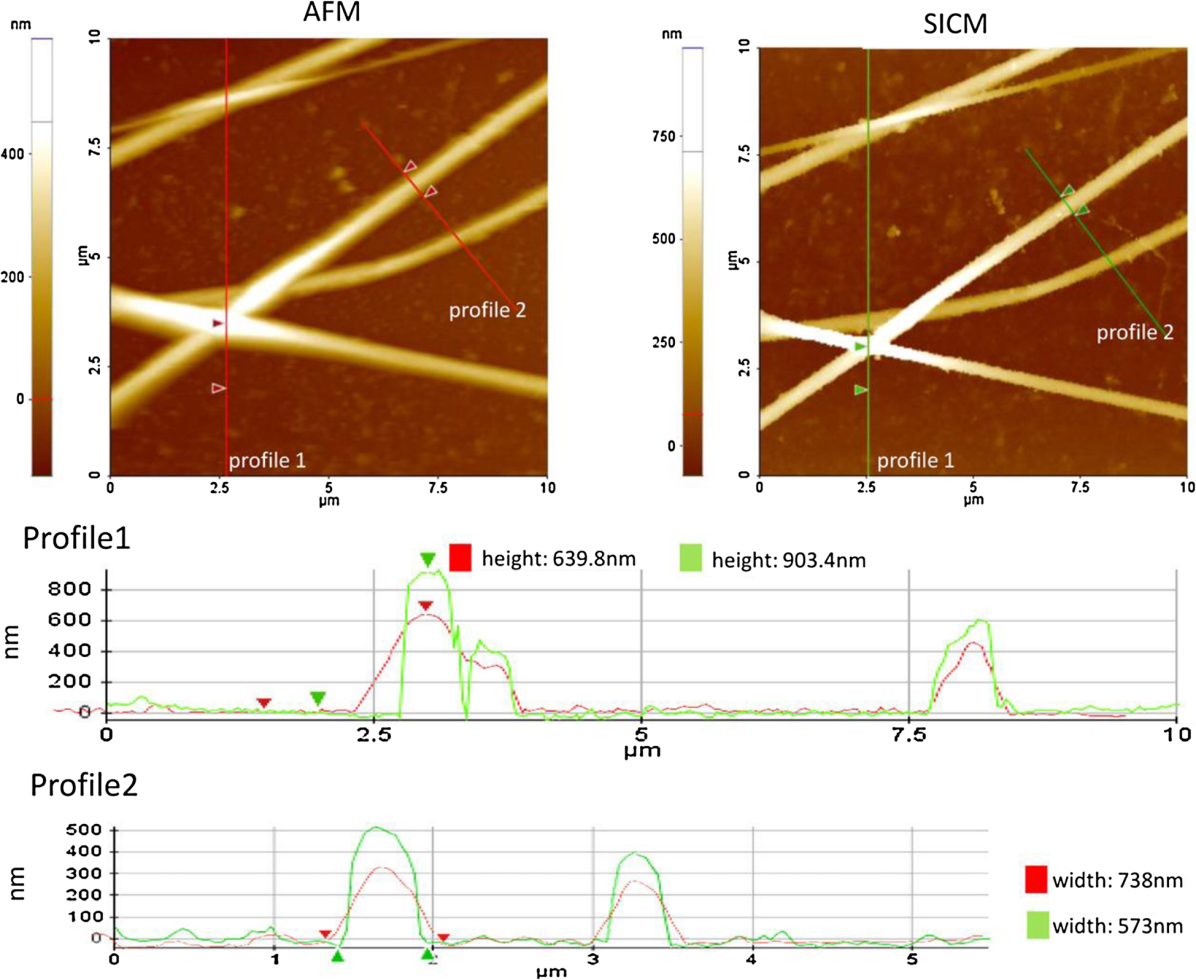
**Comparison of SICM and AFM imaging capabilities.** Collagen fibrils on a glass slide were first imaged by AFM (upper left image) and then by SICM (upper right image). Section profiles 1 and 2 are based on lines labeled “profile 1” and “profile 2” in the two upper images. From Ref. [62], copyright@2012 Elsevier.

Recently, Ushiki et al. improved the capability of hopping-mode SICM [62]. They demonstrated that SICM can visualize filaments on cell better than atomic force microscopy (AFM). Figure 5 shows AFM and SICM images of the same collagen fibrils. The height of the collagen fibrils in the SICM image is 1.4 times that in the AFM image, suggesting that SICM is better suited for non-contact imaging than AFM. The width of the collagen fibrils smaller in the SICM image is 0.7 times that in the AFM image. Because collagen fibrils are cylindrical, the width was likely influenced by the shape of the probing chip. Their finding suggests that the shape of the collagen fibrils is well-delineated in the SICM image even though the inner radius of the pipette, approximately 100 nm, was much larger than the AFM probing tip radius. The approach-retract scanning (ARS)/hopping mode of SICM is especially powerful for imaging samples with steep slopes [61]. Ushiki et al. showed that the lateral resolution of the approach--retract--scanning/hopping SICM mode is around 50 nm, which is in agreement with the values mentioned in a previous study [61]. However, the actual resolution of SICM imaging is under discussion [63].

Concurrent imaging using SECM and SICM has also been developed. Comstock et al. demonstrated the successful fabrication and application of integrated SECM--SICM nanopipette probes [64]. Approach curves for conducting and insulating substrates demonstrate the suitability of these probes for SICM and SECM with the ac redox current showing enhanced surface sensitivity. Integrated SECM--SICM imaging in both feedback- and substrate generation/tip collection modes has been demonstrated, with spatial resolution in the deep submicrometer regime. A SECM--SICM integrated system extends the capabilities of conventional SICM imaging to the characterization of electrochemical phenomena, thus enabling new applications of SICM for biological and electrochemical imaging.

### Ion-selective nanopipettes

An ion-selective electrode (ISE) with polymer membrane is a universal and known tool for the recognition of ions in a liquid, and various types of ISEs have been developed [65,66]. However, the typical size of a conventional ISE is on the order of millimeters, and thus, such as an ISE cannot be used with a nanopipette. Therefore, we developed a method to prepare an ion-selective membrane in a nanopipette; further we developed a nanopipette-based detection system for subtle signals [67–70]. The ion detection mechanism of an ISE in a nanopipette is discussed in our recent paper [71]. We demonstrated that target ions are detected by choosing the inner liquid between the ISE and wire electrode with target ion electrolyte, whereas complementary signals are obtained by replacing the inner liquid with water.

**Figure 6:**
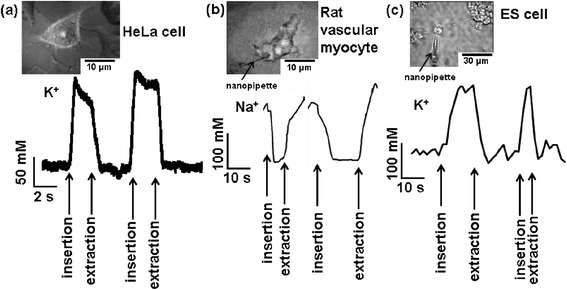
**Observations of local ion concentrations in- and outside living cells. (a)** Potassium ion concentration in- and outside HeLa cell. **(b)** Sodium ion concentration in- and outside rat vascular myocyte. **(c)** Potassium ion concentration in- and outside motor neuron cell from ES cell. The optical microscopy image of the corresponding cell is shown in the upper part in each figure. Each lower part shows the time chart of the relative concentration, and the times to insert or extract the nanopipette are pointed with arrows. From Ref. [73], copyright@2013 World Scientific.

Further, we applied our method to the determination of local concentrations of target ions in various kinds of living cells. We applied this method to determine the local concentration of potassium or sodium ions in HeLa cells [72], rat vascular myocytes, and neuron cells from ES cells. Relative differences of approximately 100 mM in the concentrations of these ions were observed in the living cells. Figure 6 shows the images and results for local ion concentrations in- and outside cells [73].

### Functional nanopipettes

Adding functionalities, including ion selectivity we developed [67–73], to nanopipettes enhance their capability for injection and detection in a normal way. Nanopipettes can include specific recognition elements for analytical discrimination based on size, shape, and charge density. Further, a fully electrical read-out and easy and low-cost fabrication are unique features that give this technology an enormous potential [7].

Carbon nanopipettes overcome the problems associated with electrical measurements during the injection process [74]. Schrlau et al. demonstrated a fabrication process for carbon nanopipettes without any assembly [75]. Carbon nanopipettes can penetrate cell membranes and controllably inject minute material quantities into the cells without hindering cell growth and without breaking the cells. The hollow conductive carbon film in carbon nanopipettes allows the concurrent use of these nanopipettes as injectors and electrodes. Moreover, carbon nanopipettes can be used as nanoscale biosensors by functionalizing their carbon surface with proteins and/or oligonucleotides. Since the carbon walls of these nanopipettes are transparent to electrons, we propose that carbon nanopipettes can be used as sample holders for electron microscopy. In a future study, measuring cell response such as changes in membrane potential during injection and developing massive carbon nanopipette arrays that can concurrently interact with a large number of cells can be envisioned.

Nogawa et al. fixed an organic lipid nanotube with an inner diameter of 50 nm on the inside of a glass micropipette with an inner diameter of 1800 nm by using a three-dimensional micromanipulation technique [76]. They sealed the interspace between the lipid nanotube and glass micropipette with a photocrosslinkable resin in order to fix the lipid nanotube onto the micropipette. A fluorescent solution of rhodamine 6G was applied such that it would be spouted by an electroosmotic flow caused by the Coulomb force. When the applied voltage was 426 V, the initial spout of the solution from the tip of the lipid nanotube to the nanopipette was observed using fluorescent microscopy. The spout profile was in remarkable contrast to that obtained for a lipid-nanotube-free glass micropipette in terms of the volume spouted. A theoretical estimation allowed to prove that the newly developed nanopipette will be able to spout attoliter volumes of solutions. This is equivalent to 1/1000—1/100 of the amount that can be dispensed by commercial pipette injection systems.

Multi-barrel nanopipettes can be used for simultaneous observation and manipulation. Seger et al. demonstrated such a technology for semi-automated, high-viability cell injection [77]. Their double-barrel voltage-controlled nanoinjection system addressed the need to deliver material into a cell controllably, reliably and without the need for highly specialized manual operations. Their cell injection system, based on SICM technology to locate the cell surface and inject an arbitrary material into a single cell, demonstrated the robustness and speed required for efficient and effective research in single-cell biology. Multi-component injection with a single nanopipette is a potential technology for injecting a customized cocktail of molecules into an individual cell, and is limited only by the number of barrels in the nanopipette. Takahashi et al. developed a simple, affordable, and quick method for fabricating a double-barrel carbon nanoprobe for functional nanoscale electrochemical imaging by using SICM distance feedback control [78]. Using this fabrication method, they prepared probes with controllable radii in the range of 10 nm to 1 mm. Their developed probes allowed the mapping of neurotransmitter release sites together with associated changes in cell topography that occur during exocytosis. In future, this technique can be extended to perform intracellular measurements.

## Perspective on nanopipettes

**Figure 7:**
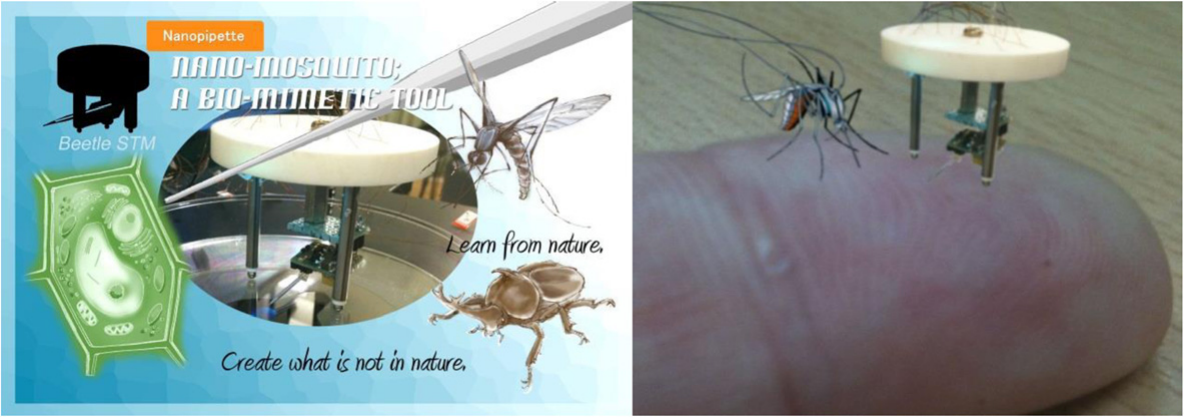
**Left figure: schematic showing the concept of a “** ***nano-mosquito*** **”; three-legged beetle-type robot with a nanopipette.** The design of the nano-mosquito is inspired from the beetle STM developed by Besocke. The quote “Learn from nature and create what is not in nature” is by Dr. Heinrich Rohrer who invented STM. See Ref. [79] for more details on the nano-mosquito. Lower figure: conceptual image of the nano-mosquito reduced to the millimeter scale. Both illustrations were partly prepared by Miyuki Miyata.

The position and insertion/extraction motion of a nanopipette is usually controlled by motors and piezo actuators. Recently, we fabricated a robot called a “nano-mosquito”—a three-legged piezo robot with a nanopipette, as shown in Figure 7 [79]. This robot was developed on the basis of Besocke-type scanning tunneling microscopy (STM) [80]. The diameter of the the robot was approximately 70 mm; this value should be reduced for inserting and extracting the nanopipette with high accuracy and stability, as well as for reducing the Q-factor of the robotic system.

Placing nanotubes on the outside of a nanopipette provides a new functional tool. Kim and Lieber fabricated nanotube nanotweezers by attaching two nanotubes on a pulled glass micropipette [81]. The free ends of the nanotubes could be closed and opened by applying voltages to the electrodes. The mechanical capabilities of these nanotweezers were demonstrated by using them for grabbing and manipulating submicronsized clusters and nanowires. Further, the conducting nanotube arms of the tweezers were also used for measuring the electrical properties of nanoclusters and nanowires.

Recently, carbon nanotube pipettes have been attached to the top of a glass pipette. Singhal et al. successfully fabricated a multiwalled carbon nanotube endoscope at the tip of a glass pipette; this endoscope can probe intracellular environment with a spatial resolution of approximately 100 nm and can access organelles without disrupting the cell [82]. Moreover, the endoscope can be remotely maneuvered to transport magnetic nanoparticles and attolitre volumes of fluids to and from precise locations. Further, the endoscope can fit standard instruments and can thus be used in a broad range of applications such as minimally invasive intracellular probing, drug delivery, and single-cell surgery.

The use of nanopipettes in vacuum will help in elucidating catalyst reactions. In particular, a mass spectrometer equipped with a nanopipette--named scanning mass analysis probe (SMAP)--will enable the determination of the kind of species under the pipette by socking out the species. This method will provide direct evidence for the location of catalytic reaction, whether it occurs at surface edge or terrace. Very recently, Yuill et al. used a nanopipette as an electrospray ionization emitter for mass spectrometry [83]. This application involves nanopipette geometry opposite to that proposed by us; they set the mass spectrometer at the top of a nanopipette [83], whereas we set it at the entrance of the pipette in our proposed SMAP.

Non-linear oscillations in the ionic current flowing through a nanopipette occur at a certain diameter at which the Debye length of the double layer, consisting of Stern and Gouy-Chapman layers, is comparable to the inner nanopipette radius [38]. In addition ICR occurs in nanopipettes [28]. These phenomena impede the proper observation of ionic currents by using nanopipettes. Therefore, the modification of the inner nanopipette using polymers such as polyethyleneimine was investigated [84]. The hydrophobization of the inner nanopipette wall with trimethylsilyl chloride hexamethyldisilazane [85] might solve the problems due to the double layer.

**Figure 8:**
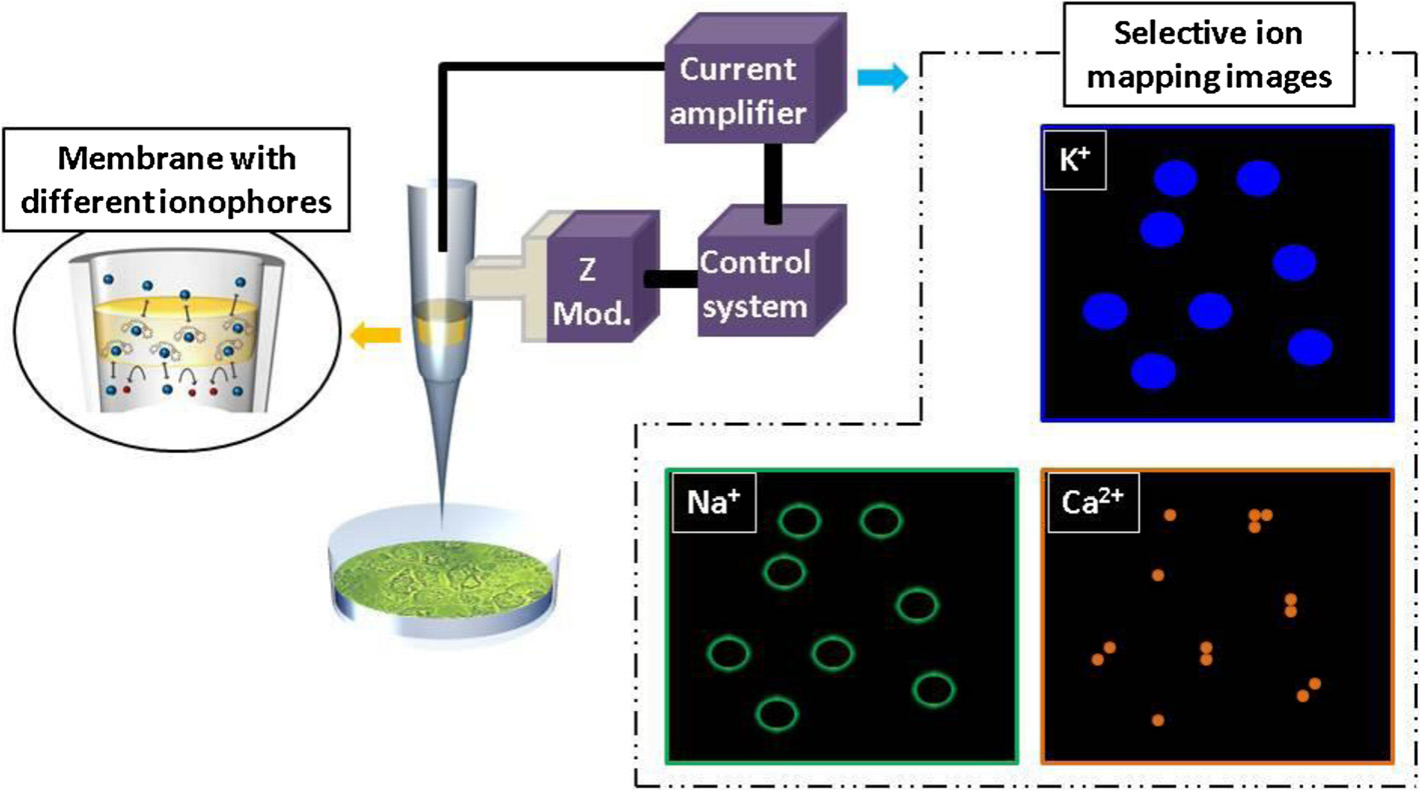
**Schematic showing ion-selective SICM withthe ion-selective nanopipette developed by us (left) and expected ion-selective SICM images (right).**

Ion-selective nanopipettes can be used for SICM, *i.e*., ion-selective SICM, as shown in Figure 8. Though SICM is commercially available [86], total ion currents are used to obtain SICM images. Ion mapping can be performed by combining SICM with the ISE nanopipettes developed by us, this technique will elucidate the dynamics of ion channels in cells by the real-time observation of targeted ions infusing in and extracting out of the ion channels. Ion-selective SICM will also provide information on localized ions at liquid–solid interfaces and will elucidate electrochemical reactions occurring at electrodes. We are developing ion-selective SICM for greater exploration of the nanoworld than is possible using conventional SICM.

## Conclusion

The applications of nanopipettes have been reviewed in this paper. Nanopipettes are used to transfer materials such as gold nanoparticles, chemical compounds, and proteins. Moreover, they can be used for nanoscale imaging, especially for observing biological materials because their interaction with the observation samples is softer than that in the case of conventional AFM. Further, we have demonstrated that a nanopipette can be afforded the functionality to select ions such as those of sodium, potassium and calcium by fabricating an ion-selective membrane in the nanopipette. The fabricated ion-selective nanopipettes can be used to determine local ion concentrations in- and outside living cells. We have also predicted the emergence of nanopipette applications such as SMAP and ion-selective SICM in future studies.

## Abbreviations

AFM: Atomic force microscopy
ES cell: Embryonic stem cell
HeLa cell: Immortal cancer cell from Henrietta Lacks
ICR: Ion current rectification
ISE: Ion-selective electrode
SECM: Scanning electrochemical microscopy
SERS: Surface enhanced Raman spectroscopy
SICM: Scanning ion conductance microscopy
SMAP: Scanning mass analysis probe
STM: Scanning tunneling microscopy
